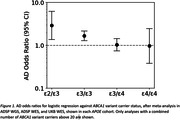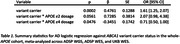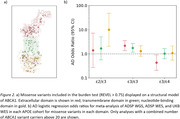# *ABCA1* rare variants interact with *APOE* isoforms to confer Alzheimer’s Disease risk

**DOI:** 10.1002/alz.092053

**Published:** 2025-01-03

**Authors:** Andrés Peña‐Tauber, Ricardo Hernandez Arriaza, Yann Le Guen, Chaitan Khosla, Michael D Greicius

**Affiliations:** ^1^ Stanford University School of Medicine, Stanford, CA USA; ^2^ Stanford University, Stanford, CA USA; ^3^ Stanford University, School of Medicine, Stanford, CA USA

## Abstract

**Background:**

Several lines of evidence now suggest that *ABCA1* plays a role in Alzheimer’s disease (AD). Rare variants on *ABCA1* increased AD risk in a large genetic study, and mouse models suggest that increasing ABCA1 activity can reverse signs of AD pathology. While there is growing consensus that ABCA1 and ApoE directly interact, it is unclear how *APOE* genotype affects this interaction in the context of neurodegeneration.

**Method:**

We conducted burden tests of rare variants (MAF < 1%) on *ABCA1* either causing loss of function or having a REVEL score above 0.75 in European‐ancestry participants from ADSP whole‐genome (WGS), whole‐exome (WES) sequencing, and UK Biobank WES datasets. All analyses were adjusted for sex, PCs 1‐4, and sequencing platform. Stratifying by *APOE* genotype, we tested the association between *ABCA1* variant carrier status and AD using logistic regression, then meta‐analyzed the results with fixed‐effect inverse‐weighted variance. To test directly for an *APOE* interaction with *ABCA1*, we analyzed all *APOE* genotypes together adding interaction terms *ε*2*ABCA1 and *ε*4*ABCA1, and additionally adjusted for *APOE ε*2/*ε*4 dosages.

**Result:**

Carrying an *ABCA1* variant was associated with increased AD risk in *APOE ε*2*/ε*3 and *ε*3*/ε*3 cohorts. In contrast, the association was not significant in *ε*3*/ε*4 or *ε*4*/ε*4 (**Fig. 1**). Models including cross‐terms in the whole‐*APOE* cohort supported an interaction between *ABCA1* variants and *APOE ε*4 dosage diminishing risk compared to having an *ABCA1* variant by itself (**Table 1**). Analysis of missense variants by themselves showed a significant protective effect for extracellular domain mutations in *ε*3*/ε*4, whereas they increased risk in *ε*3*/ε*3 and showed no effect in *ε*2*/ε*3 (**Fig. 2**). Conversely, *ε*2*/ε*3 saw a risk‐increasing association only with nucleotide‐binding domain mutations.

**Conclusion:**

*ABCA1* rare variants are associated with AD risk and the effect size appears to be modulated by *APOE* genotype, showing significant interaction with *ε*4 dosage. This suggests an interplay between ABCA1 and different ApoE isoforms in contributing to AD, highlighting a potential pathogenic mechanism. Further, as extracellular domain mutations show diverging impacts on AD risk depending on *APOE* genotype, this domain may play a critical role in the pathogenic interaction between ABCA1 and ApoE.